# Components of Physical Activity and Their Relationships with Quality of Life and Cancer-Related Fatigue in Breast Cancer Survivors in Malaysia

**DOI:** 10.21315/mjms-12-2024-996

**Published:** 2025-06-30

**Authors:** Aiman Nadia Akmar Rahman, Foong Kiew Ooi, Maria Justine, Mohd Nidzam Jawis, Maya Mazuwin Yahya

**Affiliations:** 1 Exercise and Sports Science Programme, School of Health Sciences, Health Campus, Universiti Sains Malaysia, Kelantan, Malaysia; 2 Centre for Physiotherapy Studies, Faculty of Health Sciences, Universiti Teknologi MARA Selangor, Puncak Alam Campus, Selangor, Malaysia; 3 Department of Surgery, School of Medical Sciences, Health Campus, Universiti Sains Malaysia, Kelantan, Malaysia

**Keywords:** breast cancer survivors, physical activity, cancer-related fatigue, quality of life, physical well-being, functional well-being

## Abstract

**Background:**

The main aim of the study was to determine the relationships between components of physical activity level with quality of life (QoL) and cancer-related fatigue (CRF) among breast cancer survivors in Malaysia.

**Methods:**

Eighty-three female breast cancer survivors with mean age 52.8 (8.7) years old participated in this study. The participants were asked to answer Short Form International Physical Activity Questionnaire (SF IPAQ) to identify physical activity level, Breast Cancer Functional Assessment of Cancer Therapy (FACT-B) questionnaire to determine the QoL, and Brief Fatigue Inventory (BFI) scale questionnaire to assess CRF. Descriptive statistic and Pearson correlation were performed for statistical analysis.

**Results:**

There was a significant positive correlation between time spent for moderate to vigorous physical activity (mins/week MVPA) and functional well-being, a component of QoL (*r* = 0.217, *P* = 0.049). There was a significant negative correlation between the days spent for moderate physical activity (days/week MPA) and the severity of fatigue, a component of CRF (*r* = −0.234, *P* = 0.033). A negative correlation between total CRF score and total QoL score (*r* = −0.298, *P* = 0.01) was also observed.

**Conclusion:**

Participation in physical activity might improve QoL and reduce the severity of CRF in Malaysian breast cancer survivors.

## Introduction

Breast cancer is a major health problem worldwide including Malaysia, with a rising incidence rate which impacts the quality of life (QoL) of breast cancer survivors. In Malaysia, breast cancer is ranked as the top or highest newly diagnosed cancer case (17.3%) and reported as the second cause of cancer death (11.0%) compared to other cancer cases (1). Survival information is an important predictor on the effectiveness of cancer diagnosis and treatments, which reflects the quality of care for cancer patients. A study conducted in Malaysia showed that in the last two decades, the five-year survival rate for those diagnosed with breast cancer was only 49% (2).

Mishra et al. reported that involvement in physical activity positively affects the physiological changes, improving the QoL of breast cancer survivors in terms of body image, self-esteem, sleep disturbance, and social function (3). In addition, Kim et al. reported that engaging in physical activity among breast cancer survivors was significantly associated with decreased mortality and increased physiological functions; the major health outcomes were increased cardiorespiratory functions, muscular strength, and immune cell components (4). Unfortunately, according to Lee et al., breast cancer survivors in Malaysia had inadequate physical activity levels at diagnosis, which further decreased over time (5).

Cancer-related fatigue (CRF) is one of the major problems among cancer survivors (6), which is described as a distressing permanent feeling of tiredness or exhaustion (7). Fatigue levels are high during chemotherapy and at the end of the chemotherapy session, maintaining after one year and only declining up to ten years for cancer survivors (8). CRF is multifactorial and can be influenced by various demographic, medical, psychosocial, behavioural, and biological factors (9). Additionally, CRF is partly induced by cancer treatments such as chemotherapy, radiation, and endocrine therapy, which can be exacerbated by physically inactive lifestyle (6).

CRF causes survivors to depend on others for home management, transportation, and even self-care activities, which are demoralising and discouraging. Furthermore, CRF forces patients to engage in unwanted activities in an attempt to cope with their fatigue, such as lying down or taking naps, which indirectly leads to physical decondition. Mustian et al. mentioned that CRF is distressing and has a great negative impact on daily activities and QoL among cancer survivors (10). A study carried out by Hirschey et al. reported that cancer survivors who followed the prescribed exercise programme gained benefits of 40% lower mortality risk and decreased fatigue level (11).

QoL refers to a person’s perception and satisfaction with life and their general appraisal of their level of functional well-being (12). Breast cancer diagnosis has been shown to have an impact on physical, mental, economic, as well as social and lifestyle. Breast cancer survivors have gone through challenging experiences which affect their physical, emotional, and social well-being (13). The findings on the QoL among breast cancer patients have been reported to be inconsistent across studies. With changing norms, values, lifestyle, as well as conceptualisation of QoL that keep on changing over time, more current studies on QoL in breast cancer survivors are warranted.

Existing research on breast cancer in Malaysia has primarily focused on screening practices, awareness, and diagnostic delays. However, the experiences and QoL among breast cancer survivors have not been adequately explored. Hence, exploring the QoL among breast cancer survivors in the Malaysian context can provide valuable insights into the unique challenges they face and the development of targeted interventions to improve their well-being and support their reintegration into society.

Engagement in physical activities is a nonpharmacological and low-cost approach to enhance the overall health status of an individual. Involvement in physical activity among cancer survivors should be encouraged for them to cope with the severity of fatigue and to improve QoL. To date, information on the relationships between physical activity level, QoL, and CRF in Malaysian breast cancer survivors are limited. In this study, the components of physical activity level among Malaysian breast cancer survivors were assessed, and their relationships with QoL and CRF were determined. The findings of this study are expected to provide new clinical evidence for healthcare professionals to enhance the management of cancer rehabilitation.

## Methods

### Study Design and Participants Recruitment

This study applied a cross-sectional study design. A total of 83 participants were selected using a purposive sampling method according to the inclusion and exclusion criteria set for this study. To be eligible for the study, participants were required to comply with the following inclusion criteria: Malaysian females diagnosed with breast cancer (stages 1–4) for more than one year prior to data collection and have completed primary treatment. In addition, these females should be 25 years of age or older and possess normal cognitive function and communication abilities. Those with a history of other types of cancer were excluded from the study.

All participants who agreed to participate in the study gave written informed consent. Participants were recruited from two hospitals and one primary clinic in Malaysia. The two hospitals involved were Breast Cancer Awareness and Research (BesTARI) Unit of Hospital Universiti Sains Malaysia, Kubang Kerian, Kelantan, Malaysia and Institut Kanser Negara, Putrajaya. Klinik Hayat, Jasin Bestari, Melaka was the primary clinic involved in this study.

Data collection procedures were conducted by the researcher at the above-mentioned hospitals and primary clinic. During the visit, participants were required to complete a physical activity level assessment by answering the Short Form International Physical Activity Questionnaire (SF IPAQ) questionnaire. The Brief Fatigue Inventory (BFI) scale questionnaire and the Breast Cancer Functional Assessment of Cancer Therapy (FACT-B) questionnaire were also required to be answered by the participants to assess their level of fatigue and QoL, respectively. All questionnaires were available in English and Malay versions. Participants were given the option to choose their preferred language version based on their comfort and proficiency.

### Sample Size Calculation

The sample size used in this study was calculated by using Gpower 3.1.9.4 software. The power of study was set at 80%, and the alpha level was set at 5%. The effect size of magnitude of Pearson correlation was set at medium size of *P* = 0.30. The result of Gpower showed that the number of participants to be recruited was 84. By considering a 10% dropout rate, 92 participants were required to be recruited in the study. Nevertheless, nine of the participants withdrew from the study due to personal reasons. Hence, a total of 83 participants completed this study.

### Assessment of Physical Activity Level

The Short Form International Physical Activity Questionnaire (SF IPAQ) was used to determine the physical activity level of the participants. SF IPAQ assesses physical activity domains, which include walking, moderate intensity activities (MPA), moderate-vigorous activities (MVPA), and vigorous intensity activities (VPA), as well as sitting which indicates sedentary activity. SF IPAQ consists of eight items to estimate the time spent on performing physical activities (moderate to vigorous) and inactivity (time spent sitting). Details of the items are illustrated in Table 1. The IPAQ demonstrated good reliability and validity for evaluating physical activity among the Malaysian population (14). The components of physical activity level consisted of days, time in minutes, and energy (MET) spent per week for VPA, MPA, MVPA, walking, and sitting.

In the present study, the data collected were processed and analysed based on the IPAQ guidelines for data processing and analysis and scoring protocol (www.ipaq.ki.se). The SF IPAQ questionnaire has been validated by Teh et al., who reported that the Malay version demonstrated good reliability and validity (15). Craig et al. have validated the international version in 12 countries (16).

### Assessment of QoL

QoL was assessed using the Breast Cancer Functional Assessment of Cancer Therapy (FACT-B) questionnaire. The FACT-B was developed by the Functional Assessment of Chronic Illness Therapy authority (FACIT.org) to measure the QoL among breast cancer survivors. The tool consisted of five subdimensions, which are physical well-being (PWB), social/family well-being (SWB), emotional well-being (EWB), functional well-being (FWB), and a subscale on additional concerns for breast cancer survivors (BCS). The details of items of the subdimensions are illustrated in Table 2.

Responses were recorded on a 5-point Likert scale ranging from 0 (not at all) to 4 (very much). The total score of FACT-B varies from 0 to 148, and a higher score indicates a better health-related QoL. A validation study by Md Yusof et al. demonstrated that the Malay version of FACT-B possesses strong internal consistency, with an overall Cronbach’s α value of 0.88 and domain-specific values ranging from 0.62 to 0.88 (17). This confirms that the Malay version is both reliable and suitable for assessing QoL among Malaysians.

### Assessment of CRF

BFI scale questionnaire was used to measure fatigue levels among the participants in this study. The BFI scale questionnaire was developed by Mendoza et al., with internal consistency reported at 0.96 (18). It consisted of nine items, with 0 to 10 numeric rating scales. Three items require participants to rate the severity of their fatigue at its “worst,” “usual,” and “now” during normal waking hours, with 0 being “no fatigue” and 10 being “fatigue as bad as you can imagine.” Six items assess the amount that fatigue has interfered with different aspects of the participant’s life during the past 24 hours. The details of the items in the BFI are presented in Table 3.

The Malay version of the BFI has been linguistically validated. The validity and reliability of the BFI questionnaire have been reported by Paramita et al. (19) and have been applied to the Malaysian population (20).

### Data Analysis

Statistical Package for Social Sciences (SPSS) version 24.0 was used for the statistical analysis. Descriptive data are presented in frequency, percentage (%), and mean (standard deviation [SD]). Correlation analysis was conducted to evaluate the magnitude and direction of the linear relationship between the variables by using Pearson correlation. A *P*-value of < 0.05 was considered as statistically significant.

## Results

Eighty-three breast cancer survivors diagnosed with either cancer stages 1, 2, 3, or 4 completed this study. The age of the participants ranged from 35 to 72 years, with a mean age of 52.8 (8.7) years.

### Components of Physical Activity Level of Participants

Table 1 shows the results on the components of physical activity level for the participants obtained via the SF IPAQ questionnaire. The mean total physical activity was 2,043.45 (3,423.46) MET-minutes per week. Additionally, the mean time spent for moderate intensity activity was 165.90 (300.64) min per week, the mean time spent was 80.54 (317.58) min per week for vigorous intensity physical activity, and the mean time spent for moderate-vigorous physical activity was 246.45 (533.17) min per week with the highest value among three types of physical activity level. It was also observed that the mean walking time of the participants was 275.24 (399.15) min per week, and the mean sitting time of the participants was 218.25 (123.40) min per week.

### QoL of Participants

The results for the QoL of breast cancer survivors assessed by the FACT-B questionnaire are illustrated in Table 2. The mean total score of QoL was 110.44 (2.68). The mean total scores of the subscales for physical, social, emotional, functional well-being, and additional concerns were 20.55 (0.83), 23.84 (0.68), 18.15 (0.67), 23.2 (0.67), and 25.11 (0.79), respectively. Among the subscales, the emotional subscale showed the lowest score, whereas the participants scored highest in the additional concern subscale.

### Profile of CRF of Participants

The profile of CRF of the participants, as evaluated using the BFI questionnaire is presented in Table 3. Based on the findings, 77.1% of breast cancer survivors experienced CRF, whereas 22.9% of breast cancer survivors did not experience CRF. The mean of the nine BFI items, i.e., total CRF score was 3.20 (2.69), which can be categorised as mild level of CRF. The mean for the item regarding “fatigue at its worst” which indicates the severity of CRF, was 4.34 (3.09) with mode value of 5.

### Correlations of Components of Physical Activity Level with QoL

The correlations of components of physical activity level with QoL components are presented in Table 4. There was a statistically significant negative correlation between days spent for MPA and physical well-being (*r* = −0.226, *P* = 0.04) and a statistically significant negative correlation between days of walking per week and functional well-being (*r* = −0.264, *P* = 0.016). However, a significant positive correlation was observed between time spent for MVPA and functional well-being (*r* = 0.217, *P* = 0.049).

### Correlations of Components of Physical Activity Level with CRF

Regarding the correlation between physical activity level variables and components of CRF, a negative linear relationship was found between the severity of fatigue and the number of days spent engaging in moderate physical activity (*r* = −0.234, *P* = 0.033). Other measured parameters did not show any significant relationships.

### Correlations between QoL and CRF

All QoL components, i.e., physical well-being (*r* = −0.332, *P* = 0.002), social well-being (*r* = −0.249, *P* = 0.023), functional well-being (*r* = −0.280, *P* = 0.010), additional concerns (*r* = −0.246, *P* = 0.025), total QoL (*r* = −0.298, *P* = 0.006), except emotional well-being subscale, were statistically significant and negatively correlated with total CRF score (Table 5).

## Discussion

The mean times spent for moderate intensity physical activity (165.90 min per week) and vigorous intensity physical activity (80.54 min per week) of the participants in this study (Table 1) have met the requirement and recommendation of the American Cancer Society, i.e., at least 150 min per week of moderate intensity and or 75 min per week of vigorous intensity physical activities (21). These findings imply that the participants engaged in a substantial amount of physical activity, which is believed to be beneficial for overall health and survivorship outcomes.

It is generally known that sedentary behaviour can negatively impact health. Reducing prolonged periods of sitting and performing more movements throughout the day may be beneficial for overall health in breast cancer survivors. This study found that participants spent more time engaging in “moderate to vigorous physical activity” (246.45 min per week) compared to “moderate” and “vigorous” physical activity, respectively. It was also found that the walking time of 275.24 minutes per week was higher than the mean sitting time of 218.25 minutes per week. These findings indicated that the breast cancer survivors of this study had a high level of “moderate to vigorous” physical activity and relatively low sitting time.

The QoL of the breast cancer survivors assessed by the FACT-B questionnaire showed that the mean total score of QoL was 110.44 (2.68), i.e., 74.62 % of 148 (the maximal total QoL score) (Table 2). The higher score on the FACT-B indicates a better QoL among breast cancer survivors. Therefore, the mean total score of QoL observed in this study demonstrated that the participants had moderate to good levels of QoL. It was also evident in the present study that among the subscale domains, the emotional subscale showed the lowest score, whereas participants scored highest in the additional concern (BCS) subscale.

The result on the mean score of QoL was also reported by Fatimah et al. in their study conducted among Malaysians (22). They found that the mean score of QoL in Malaysian breast cancer survivors, measured by the EORTC QLQ-30 questionnaire, was 73.68 (17.32), with a maximum score of 100. Nevertheless, their result showed that there was a high rate of social support, i.e., subscale of social/family well-being, especially from family members. In the present study, the participants emotional subscale showed the lowest score, while the additional concern subscale showed the highest score. These findings reflect that breast cancer survivors may be burdened by perceptions and thinking about overall survival and planning for the children’s future instead of thinking about themselves, as mentioned by Heidary et al. (23).

The present study found that 77.1% of breast cancer survivors experienced mild level of CRF with mean BFI score of 3.20 (2.69) (Table 2). It was also found that the mean CRF score of “fatigue at its worst” was 4.34 (3.09), indicating moderate level of CRF. A previous study conducted by Muthanna et al. in Malaysia reported that 58.9% of breast cancer patients (*N* = 292) presented with CRF (24). A systematic review meta-analysis on the prevalence and risk factors of CRF conducted by Ma et al. found that the prevalence of CRF in previous studies varied from 14.0% to 100%, with the estimates of pooled prevalence of CRF in 40 developed countries at 43%, and 68% of pooled prevalence of CRF in 18 developing countries (25). Ma et al. also stated that the prevalence of CRF is lower in developed countries due to better mechanisms and policies in addressing the problem compared to developing countries (25). Álvarez-Salvago et al. found that 41.2% of fatigued breast cancer survivors reported developing persistent CRF after five years completion of cancer treatment (26).

Regarding the relationship between physical activity and QoL (Table 3), there was a statistically significant weak negative correlation between days spent for moderate physical activity (MPA) and physical well-being (*r* = −0.226, *P* = 0.04), which indicates a controversial explanation of the more days spent for MPA, there is a slight reduction in the physical well-being of the participants. In the current context, physical well-being refers to the overall physical health and functional capacity of individuals. The reason for the present result is unanticipated, since it is established that physical activity improves well-being (27). Given the controversial nature of this finding, fatigue and inappropriate types and intensity levels of MPA performed can be the reasons why MPA negatively impacts the physical well-being of breast cancer survivors.

The present study also found that there was a significant weak positive correlation between time spent on moderate to vigorous physical activity (MVPA) and functional well-being among breast cancer survivors (*r* = 0.217, *P* = 0.049). This finding is supported by a study conducted by Nurnazahiah et al. in Malaysia, who also reported that longer time spent on MVPA was associated with improved QoL, whereas sedentary behaviour was associated with lowered QoL, specifically the functioning score in the QoL domains (28).

The present study also showed a statistically significant negative relationship between the number of days spent walking and functional well-being (*r* = −0.264, *P* = 0.016), indicating that increased walking days decrease the participant’s score on functional well-being. Although walking is generally considered beneficial and improves functional well-being and QoL as reported in previous studies (27, 29, 30), our findings showed contrasting effects. The effect of walking to functional well-being varied depending on the stage of recovery, overall health, as well as differences in physical and mental states of the breast cancer survivors (30). Since this present study did not determine the type and intensity of walking among breast cancer survivors, future research using objectively measured equipment, such as an accelerometer, is warranted.

This study demonstrated that there was a significant negative weak relationship between the severity of fatigue and days spent for MPA (*r* = −0.234, *P* = 0.033). This was probably attributed to the higher level of fatigue, and thus, less days were spent for moderate physical activity by the participants. Liu et al. mentioned that selected exercise programmes, such as yoga and resistance exercise, can lower cytokine-related transduction via physiological mediation and thus lead to reduction in CRF (31). A study conducted by Swen et al. among breast cancer survivors of Black women found that the association between physical activity and fatigue was strongest among women aged under 50 years (32). Meanwhile, Nilson et al. (33) found that younger women who met physical activity guideline by the American Cancer Society (21) – which is 150 minutes of MVPA per week – have higher physical activity but lower levels of fatigue compared to those who did not meet the guideline. For women aged over 50 years, Nilson et al. found no significant difference in fatigue scores based on physical activity levels (33).

In contrast to the results of the current investigation, a study by Alvarez-Bustos et al. among 180 breast cancer survivors found that weekly physical activity, measured by accelerometers at moderate, vigorous, or sedentary levels, was not associated with CRF scores (34). The authors hypothesised that their participants were already physically active, with an average of more than 250 minutes per week spent on MVPA prior to treatment. Hence, the physical activity levels no longer influence their fatigue level.

The results displayed in Table 5 showed that QoL components, i.e., physical well-being, social well-being, functional well-being, additional concerns, and total QoL scores, were statistically significant and negatively correlated with the total CRF score, indicating that higher QoL scores were related to lower total score of CRF. In other words, breast cancer survivors with higher scores of CRF have lesser QoL. This study finding was supported by Gupta et al. (35) and Tao et al. (36), who also reported that CRF have negative effect on QoL. Our findings were in agreement with the fact that breast cancer survivors who had better QoL often experienced less fatigue, as improved physical and emotional well-being could contribute to greater energy levels and overall quality of life. Conversely, those with low QoL had increased fatigue, which could have a detrimental effect on their daily activities and general well-being.

## Conclusion

This study demonstrated that there are significant relationships between physical activity levels, QoL, and CRF among Malaysian breast cancer survivors. The study findings imply that physical activity might improve QoL and reduce the severity of CRF in Malaysian breast cancer survivors.

## Figures and Tables

**Table 1:** Results on components of physical activity level of the participants obtained from the Short Form International Physical Activity Questionnaire

Variables of physical activity level	Mean (SD)
Days/week VPA	0.72 (1.80)
Minutes/week VPA	80.54 (317.58)
Days/week MPA	2.66 (2.56)
Minutes/week MPA	165.90 (300.64)
Days/week walking	5.22 (2.16)
Minutes/week walking	275.24(399.15)
Days/week MPA + walking	7.88 (3.04)
Minutes/week MPA + walking	441.14 (537.24)
Minutes/week MVPA	246.45 (533.17)
Minutes/week MVPA + walking	521.69 (749.28)
MET-min/week VPA	555.60 (1,806.68)
MET-min/week MPA	663.61(1,202.56)
MET-min/week walking	824.23 (1,266.24)
MET-min/week total	2,043.45(3,423.46)
Minutes/week sitting time	218.25 (123.40)

VPA = vigorous physical activity; MPA = moderate physical activity; MVPA = moderate to vigorous physical activity; MET = metabolic equivalent of task

**Table 2:** Results on QoL of the participants obtained from the Breast Cancer Functional Assessment of Cancer Therapy (FACT-B) questionnaire

Items		Mean	SD
Physical well-being (PWB)			
GP1	I have a lack of energy	1.58	0.18
GP2	I have nausea	0.33	0.10
GP3	Because of my physical condition, I have trouble meeting the needs of my family	1.10	0.17
GP3	I have pain	1.55	0.18
GP5	I am bothered by side effects of treatment	1.15	0.17
GP6	I feel ill	1.00	0.16
GP7	I am forced to spend time in bed	0.71	0.15
Total score of PWB		20.55	0.83

Social/Family well-being (SWB)			
GS1	I feel close to my friends	3.24	0.16
GS2	I get emotional support from my family	3.69	0.08
GS3	I get support from my friends	3.40	0.15
GS4	My family has accepted my illness	3.84	0.06
GS5	I am satisfied with family communication about my illness	3.73	0.10
GS6	I feel close to my partner (or the person who is my main support)	3.65	0.13
GS7	I am satisfied with my sex life	2.64	0.19
Total score of SWB		23.84	0.68

Emotional well-being (EWB)			
GE1	I feel sad	1.00	0.15
GE2	I am satisfied with how I am coping with my illness	3.22	0.15
GE3	I am losing hope in the fight against my illness	0.25	0.12
GE4	I feel nervous	0.69	0.14
GE5	I worry about dying	1.33	0.21
GE6	I worry that my condition will get worse	1.55	0.18
Total score of EWB		18.15	0.67

Functional well-being (FWB)			
GF1	I am able to work (include work at home)	3.20	0.14
GF2	My work (include work at home) is fulfilling	3.07	0.97
GF3	I am able to enjoy life	3.53	0.10
GF4	I have accepted my illness	3.76	0.07
GF5	I am sleeping well	2.78	0.19
GF6	I am enjoying the things I usually do for fun	3.35	0.13
GF7	I am content with the quality of my life right now	3.47	0.10
Total score of FWB		23.2	0.67

Additional concerns (BCS)			
B1	I have been short of breath	0.49	0.14
B2	I am self-conscious about the way I dress	0.16	0.06
B3	One or both of my arms are swollen or tender	0.87	0.17
B4	I feel sexually attractive	1.49	0.18
B5	I am bothered by hair loss	0.91	0.17
B6	I worry that other members of my family might someday get the same illness I have	2.93	0.19
B7	I worry about the effect of stress on my illness	1.85	0.20
B8	I am bothered by a change in weight	1.40	0.20
B9	I am able to feel like a woman	2.76	0.17
P2	I have certain parts of my body where I experience pain	2.44	0.18
Total score of BCS		25.11	0.79

FACT-B total score (total QoL score)		110.44	2.68

FACT-B total score = Total scores (PWB) + (SWB)+(EWB) +(FWB) + Additional concern (BCS); PWB = physical well-being; SWB = social/family well-being; EWB = emotional well-being; FWB = functional well-being; QoL = quality of life. Additional concern (BCS) = additional concerns for breast cancer survivors.

**Table 3:** Results on profile of CRF of the participants obtained from the BFI scale questionnaire

No.	Items	Mode	Mean	SD
	aThroughout our lives, most of us have times when we feel very tired or fatigued. Have you felt unusually tired or fatigued in the last week?			
	Yes, *n* = 64 (77.1%)			
	No, *n* = 19 (22.9%)			
1.	Please rate your fatigue (weariness, tiredness) that best describes your fatigue right NOW	0	2.75	2.29
2.	Please rate your fatigue (weariness, tiredness) that best describes your USUAL level of fatigue during past 24 hours	5	3.42	2.46
3.	bPlease rate your fatigue (weariness, tiredness) that best describes your WORST level of fatigue during past 24 hours	5	4.34	3.08
	During the past 24 hours, fatigue has interfered with your:			
4.	A. General activity	0	3.52	2.63
5.	B. Mood	0	3.37	2.92
6.	C. Walking ability	0	2.92	2.66
7.	D. Normal work (includes both work outside the home and daily chores)	0	3.47	2.77
8.	E. Relations with other people	0	2.27	2.61
9.	F. Enjoyment of life	0	2.72	2.77
	BFI Cancer-related fatigue score (Total CRF score)		3.20	2.69

BFI = Brief Fatigue Inventory; CRF = cancer-related fatigue;

a This item indicates the prevalence of CRF among breast cancer survivors;

b This item indicates the severity of CRF among breast cancer survivors. The classifications of severity of CRF based on BFI score: mild = 1–3; moderate = 4–6; severe = 7–10.

**Table 4:** Correlation matrix of components of physical activity level with quality of life

Physical activity variables	Components of quality of life					

	Physical well-being	Social well-being	Emotional well-being	Functional well-being	Additional concern (BCS)	FACT-B total score
Days/week VPA	*r* = −0.105	*r* = 0.090	*r* = 0.086	*r* = 0.018	*r* = −0.032	*r* = −0.024
	*P* = 0.346	*P* = 0.417	*P* = 0.441	*P* = 0.870	*P* = 0.771	*P* = 0.828
Minutes/week VPA	*r* = 0.104	*r* = 0.171	*r* = 0.132	*r* = 0.180	*r* = 0.087	*r* = 0.160
	*P* = 0.352	*P* = 0.122	*P* = 0.232	*P* = 0.103	*P* = 0.432	*P* = 0.148
Days/week MPA	***r*** ** = −0.226** ^*^	*r* = 0.081	*r* = −0.029	*r* = 0.037	*r* = 0.063	*r* = −0.060
	***P*** ** = 0.040**	*P* = 0.468	*P* = 0.796	*P* = 0.743	*P* = 0.571	*P* = 0.593
Minutes/week MPA	*r* = −0.096	*r* = 0.137	*r* = 0.076	*r* = 0.195	*r* = 0.164	*r* = 0.085
	*P* = 0.390	*P* = 0.215	*P* = 0.493	*P* = 0.078	*P* = 0.139	*P* = 0.447
Days/week walking	*r* = −0.010	*r* = −0.171	*r* = −0.204	***r*** ** = −0.264** ^*^	*r* = −0.125	*r* = −0.210
	*P* = 0.929	*P* = 0.121	*P* = 0.064	***P*** ** = 0.016**	*P* = 0.260	*P* = 0.056
Minutes/week walking	*r* = 0.063	*r* = 0.080	*r* = 0.004	*r* = 0.149	*r* = 0.088	*r* = 0.099
	*P* = 0.572	*P* = 0.472	*P* = 0.972	*P* = 0.180	*P* = 0.430	*P* = 0.374
Minutes/week MVPA	*r* = 0.008	*r* = 0.179	*r* = 0.122	***r*** ** = 0.217** ^*^	*r* = 0.144	*r* = 0.143
	*P* = 0.945	*P* = 0.105	*P* = 0.272	***P*** ** = 0.049**	*P* = 0.193	*P* = 0.197
MET-min/week VPA	*r* = 0.011	*r* = 0.167	*r* = 0.134	*r* = 0.171	*r* = 0.096	*r* = 0.121
	*P* = 0.921	*P* = 0.131	*P* = 0.228	*P* = 0.122	*P* = 0.387	*P* = 0.274
MET-min/week MPA	*r* = −0.096	*r* = 0.137	*r* = 0.076	*r* = 0.195	*r* = 0.164	*r* = 0.085
	*P* = 0.390	*P* = 0.215	*P* = 0.493	*P* = 0.078	*P* = 0.139	*P* = 0.447
MET-min/week total	*r* = 0.013	*r* = 0.167	*r* = 0.089	*r* = 0.212	*r* = 0.146	*r* = 0.134
	*P* = 0.908	*P* = 0.131	*P* = 0.424	*P* = 0.555	*P* = 0.187	*P* = 0.225
Minutes/week sitting time	*r* = −0.068	*r* = 0.100	*r* = −0.114	*r* = 0.076	*r* = −0.063	*r* = −0.009
	*P* = 0.544	*P* = 0.369	*P* = 0.306	*P* = 0.495	*P* = 0.571	*P* = 0.937

VPA = vigorous physical activity; MPA = moderate physical activity; MVPA = moderate to vigorous physical activity; MET = metabolic equivalent of task; Additional concern (BCS) = additional concerns for breast cancer survivors; QoL = quality of life. Pearson correlations (*r*) were performed to explore the relationship between measured parameters. *P* < 0.05 was considered as statistically significant. Bold numbers and ^*^ indicate statistically significant at *P* < 0.05.

**Table 5:** Correlation between QoL and CRF

Variables	Components of QoL					

	Physical well-being	Social well-being	Emotional well-being	Functional well-being	Additional concern (BCS)	Total QoL score
CRF						
Total score	***r = −*** **0.332** ^*^	***r = −*** **0.249** ^*^	*r = ****−***0.025	***r = −*** **0.280** ^*^	***r = −*** **0.246** ^*^	***r = −*** **0.298** ^*^
	***P =*** ** 0.002**	***P =*** ** 0.023**	*P =* 0.821	***P =*** ** 0.010**	***P =*** ** 0.025**	***P =*** ** 0.006**

Additional concern (BCS) = additional concerns for breast cancer survivors; QoL = quality of life; CRF = cancer-related fatigue. Pearson correlations (*r*) were performed to explore the relationship between measured parameters; *P* < 0.05 was considered as statistically significant. Bold numbers and ^*^ indicate statistically significant at *P* < 0.05.
